# F202. ABNORMAL REMODELING PROCESSING IN NEURAL GPI-APS SECRETORY PATHWAY IN SCHIZOPHRENIA

**DOI:** 10.1093/schbul/sby017.733

**Published:** 2018-04-01

**Authors:** Pitna Kim, James Meador-Woodruff

**Affiliations:** University of Alabama at Birmingham

## Abstract

**Background:**

Abnormalities in post translational modifications (PTMs) such as glycosylation have become targets of schizophrenia (SCZ) research and are implicated in the neuropathophysiology of this illness. Glycosylphosphatidylinositol (GPI) attachment to proteins and glycoproteins events; proteins essential to cellular function, including neurotransmitter rreceptors, adhesion molecules, and enzymes, are all modified by GPI. Biosynthesis of GPI -APs occurs in the endoplasmic reticulum (ER). Once GPI-APs are synthesized, they are transported from the ER to the cell surface through the Golgi apparatus. Inositol deacylation of the GPI-APs by is common PTMs. The GPI-anchored proteins (GPI-APs) play an essential role in many biological PGAP1 is required for efficient export from the ER and acts a molecular mechanism for quality control of GPI-APs. The p24 complex binds specifically to GPI-APs and plays a role in their selective trafficking by sensing the status of the GPI anchor and to promote efficient ER exit of remodeled GPI-APs. In this study, we identified abnormalities of proteins associated with the ER exit of GPI-APs in SCZ. To address mechanisms of GPI-APs ER exit, we measured expression of proteins of the GPI-APs ER exit and targeting pathway. We also measured expression of GPI-APs which have been previously implicated in SCZ, including GPC1, NCAM, MDGA2 and EPHA1.

**Methods:**

We assessed the total expression and subcellular localization of proteins involved in ER export processing of GPI-APs from the DLPFC of 15 matched pairs of SCZ and comparison subjects. Specifically, we measured levels of PGAP1 and Tmp21 (p24). Additionally, we performed a Triton X-114 phase separation to distinguish between membrane-associated and cytosolic forms of protein substrates. We confirmed the sensitivity of each target GPI-AP to phosphatidylinositol-specific phospholipase C (PI-PLC), an enzyme that specifically cleaves GPI from GPI-APs.

**Results:**

We found a significant decrease in p24 in total tissue homogenates and PGAP1 in an ER enriched fraction from subjects with SCZ. We also identified diminished sensitivity of the GPI-APs, GPC1 and NCAM, to PI-PLC treatment in SCZ.

**Discussion:**

Decreased PGAP1 in an ER enriched fraction in consistent with reduced inositol deacylation and potential dysfunction as the gatekeeper of GPI-AP ER exit in SCZ. This also suggests that the GPI-anchor is not correctly modified. Decreased p24 levels suggest downregulation of transport between the Golgi and the ER in SCZ. Additionally, we observed unchanged total level of GPI-APs in Triton X-114 phase separation, but a significant decrease in the amount of NCAM and GPC1 that was sensitive to PI-PLC in SCZ. This finding may be consistent with abnormal GPI modification of these two candidate proteins. Together, these findings suggest dysregulation of the GPI-APs remodeling system in SCZ, which may impact the structure of the GPI-anchor for SCZ-relevant proteins like NCAM and GPC1.

